# Supplementary material for: Directional Goldstone waves in polariton condensates close to equilibrium
Source: Nat Commun. 2020 Jan 10;11:217. doi: 10.1038/s41467-019-13733-x (PMC6954190; doi:10.1038/s41467-019-13733-x)
Supplement: Supplementary file 1 — Supplementary Information [file 41467_2019_13733_MOESM1_ESM.pdf]

Directional Goldstone waves in polariton condensates close to equilibrium
Supplementary Information

Dario Ballarini*,[1] Davide Caputo,[1,2] Galbadrakh Dagvadorj,[3,4] Richard Juggins,[3] Milena De Giorgi,[1] Lorenzo Dominici,[1] Kenneth West,[5] Loren N. Pfeiffer,[6] Giuseppe Gigli,[1,2] Marzena H. Szymańska,[3] and Daniele Sanvitto[1,7]

[1] *CNR NANOTEC—Institute of Nanotechnology, Via Monteroni, 73100 Lecce, Italy*
[2] *University of Salento, Via Arnesano, 73100 Lecce, Italy*
[3] *Department of Physics and Astronomy, University College London, Gower Street, London WC1E 6BT, United Kingdom*
[4] *Department of Physics, University of Warwick, Coventry CV4 7AL, United Kingdom*
[5] *PRISM, Princeton Institute for the Science and Technology of Materials, Princeton University, Princeton, NJ 08540*
[6] *Electrical Engineering Department, Princeton University, Princeton, NJ 08540*
[7] *INFN, Sez. Lecce, 73100 Lecce, Italy*

(Dated: November 4, 2019)

Supplementary Note 1: Measuring ocean wave dispersion from the air

Bathymetry, the study of floor depth in bodies of water, is a very important tool in oceanographic research, both for military and civil purposes. Satellite detection systems have recently improved our capability of measuring large areas and coasts with restricted access, but nonetheless it is worth mentioning here a previous approach which is analogous to our optical measurements. The frequency dispersion of gravity waves on the surface of intermediate-depth bodies of water (with respect to the wavelength λ) follows, according to linear wave theory, the dispersion relation $\omega = \sqrt{gk\tanh(gD)}$, where g is gravitational acceleration, D the depth of the water and $k = \frac{2\pi}{\lambda}$ [2]. Therefore, measuring the water displacement of gravity waves in space and time gives access, by simple Fourier transform, to the dispersion relation and hence to the ocean depth. Unfortunately, survey ships and arrays of sensors on the surface are extremely slow and are highly sensitive to noise, making it difficult to carry out measurements in practice. However, instead of directly measuring the whole wave structure, an alternative approach is to mount a camera on a small aircraft and take a time series of pictures of the water surface while focussed on the same geodetic position. The modulation of light diffracted by surface gravity waves over a large area allows one to extract the depth of the water from the space-time behaviour of the wave crests to a useful level of accuracy. Note that these measurements cannot be utilized to retrieve the actual wave slope magnitudes. However, this uncertainty does not affect the temporal or spatial distribution of the modulating radiance, and the dispersion relation can be obtained correctly by Fourier transforming the temporal and spatial frequencies.

In the limit of deep water, the dependence on the depth D is negligible and the previous relation reduces to $\omega = \sqrt{gk}$. While it is not directly useful for bathymetry, we report in Supplementary Fig. 1 the results of work by Dugan and co-workers published in 2001 in "Journal of atmospheric and oceanic technology" which illustrates the effect of the wind on the asymmetric population of the gravity wave dispersion [1]. The inset of Supplementary Fig. 1(a) shows a frame from the time series of images, which constitutes a cubic data set. The cubic dataset is Fourier transformed and the 2D ω-k slice through the 3D spectrum in the direction of the wind waves is shown in Supplementary Fig. 1(b). Note that if surface currents were present, a rotation of the dispersion would have been observed, corresponding to a Doppler term of the form $\mathbf{k} \cdot \mathbf{U}$, with $\mathbf{k}$ the spatial wavevector and $\mathbf{U}$ the surface current vector [3]. On the contrary, the ocean is at rest and only the excitations are moving, in the form of gravity waves, on the surface of the water. The strong, narrow peak in power on the upwind side of the spectrum with a wavelength of about 17 m ($k = 0.06\,\mathrm{m}^{-1}$) is due to the presence of a Kelvin wake generated by the boat propagating from the south-west, exciting frequencies on the opposite side of the wavevector axis. The primary wind waves populate preferentially half of the spectrum, revealing the direction of the blowing wind. In our case, the wind is replaced by density and phase fluctuations, originating in the laser spot and moving radially outward.

Supplementary Note 2: Sample and experimental setup

The semiconductor device used in these experiments is a high quality-factor ($Q > 10^5$) $\frac{3}{2}\lambda$ planar cavity with 12 GaAs quantum wells (width= 7nm) placed at the three electric field antinode positions. The front and back mirrors consist of 34 and 40 $GaAs/Al_{0.95}Ga_{0.05}As$ pair layers, respectively. The light-matter interaction, measured by the Rabi splitting (energy separation between the upper and lower polariton branches at the resonant exciton-cavity condition) is of 15 meV. We choose a sample surface area in which the detuning between the photon and

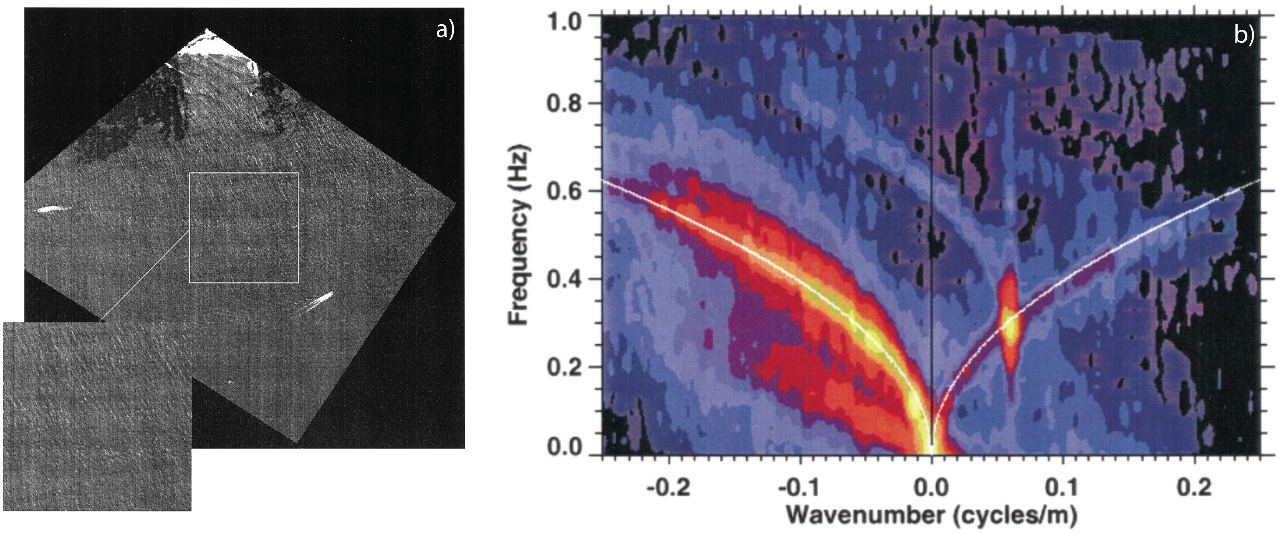

Supplementary Figure 1: **a,** Picture taken from Ref. [1] with an aerial view over Monterey Bay, near Santa Cruz, California, showing wind waves and boat wakes. In the inset, the region used to extract the wave dispersion. ©American Meteorological Society. Used with permission. **b,** Picture taken from Ref. [1] showing the cross-section along the direction of the wind (from the north-east) of the Fourier transform of the cubic data obtained from a time series of pictures in the region shown in the inset of **a**. The waves populate only the half of the dispersion corresponding to the propagation direction of the wind. The counter-propagating frequencies with positive wavevectors, visible at $\approx 0.3\,$Hz, correspond to the excitations induced by the boat, moving up-wind and generating waves only in a small range of wavelengths. ©American Meteorological Society. Used with permission.

exciton modes is about $\delta = -2\,$meV. The polariton condensation is achieved under non-resonant pumping using a single mode Ti:sapphire laser (excitation wavelength at the first minimum of the stop band, $\approx$ 735 nm, polariton emission at 774 nm). The measurements are performed in the reflection configuration using a NA= 0.5 objective for both excitation (laser spot diameter of $\approx 20\,\mu$m) and detection. The emission is sent to a spectrometer coupled with a CCD camera to visualize the energy- and spatially-resolved distribution of polaritons in real space (see Fig. 1b in the text) and, by using a 4-f configuration, in the Fourier plane (see Fig. 1a in the text). The polariton dispersion at low pumping power is shown in Supplementary Fig. 2a. Increasing the pumping power, the parabolic dispersion is hidden by the formation of a macroscopically occupied state, blueshifted in energy of $\approx 3\,$meV with respect to the bottom of the polariton dispersion (Supplementary Fig. 2b) due to the interaction with the exciton reservoir in the region below the laser spot. As shown in Fig. 1b, from the position of the laser spot, polariton are ballistically accelerated outward [4]. In Fig. 1b, the spatial region on the right of the pump spot is shown and the emission is energy resolved to allow the visualization of the high-energy expanding polariton cloud. Further increase of pump power leads to the formation of a condensate all around the pumping spot which corresponds to the bright signal at the bottom of the polariton dispersion in Supplementary Fig. 2(c-e). The pump powers used in Supplementary Fig. 2(c-e) correspond to those used in Fig. 2(a-f) but, in this case, without the spatial selection shown in Fig. 1b.

The signal is passed into a Michelson interferometer with a corner retroreflector in one arm in order to symmetrically reflect the image with respect to the central point (autocorrelation point). This allows the phase correlations between points placed symmetrically with respect to the autocorrelation point to be found in a single measurements. Repeating the same measurements with different time delays between the two arms allows one to reconstruct the whole wave dynamics. The total time range spanned in the experiments is of 200 ps and 400 ps with time steps of 1 ps and 2 ps, respectively. The sample is kept at a temperature of $10\,$K in a low-vibration cryostat, and the excitation power is chopped at a frequency of $4\,$kHz with a duty cycle of 5%.

Supplementary Note 3: Theoretical models

To understand the physical origin of the observed population of only one of the Bogoliubov branches, we investigate the system theoretically using multiple approaches. First, a numerical analysis of a homogeneous system is performed, showing the formation of the condensate and establishing that in such a system both branches of excitations are populated. Secondly, an analytical calculation

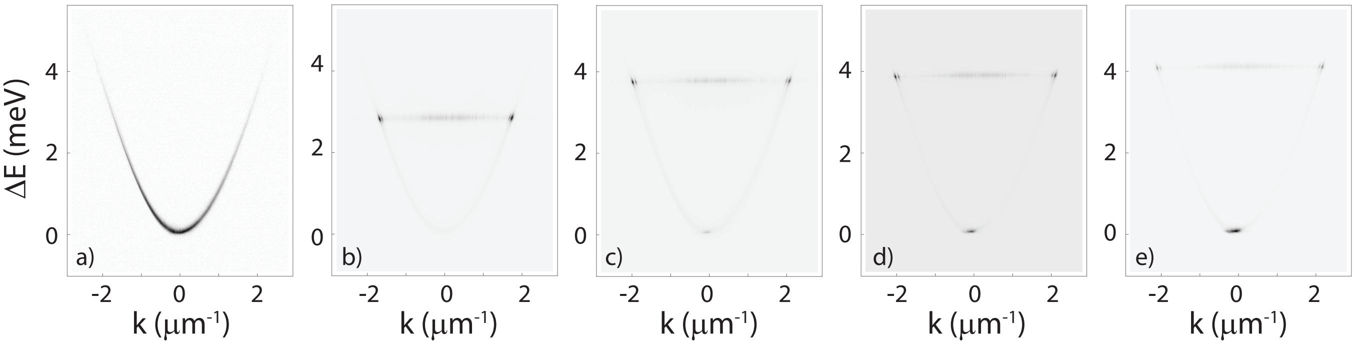

Supplementary Figure 2: Momentum space photoluminescence. **a**, At low pump powers, the parabolic dispersion is visible in a wide energy range. **b,** As the power is gradually increased, a first macroscopic occupation occurs under the laser spot at the energy set by the density in the exciton reservoir (close to $\Delta E = 3\,\text{meV}$ in **b**). Polaritons are expelled from this region creating a cloud of expanding polariton at the same energy and finite momentum. **c, d, e,** Momentum space photoluminescence for the same pumping powers used in Fig. 2. The expanding polariton cloud relaxes toward the bottom of the dispersion and form an extended condensate all around the pumping spot (k-localized signal close to $k = 0$ at $\Delta E \approx 0\,\text{meV}$). The dispersion shown here are taken without any selection, therefore averaging over different regions of the condensate.

of the photolumninescence (PL) spectrum shows that the asymmetric population is not caused by the presence of a finite condensate velocity. Lastly, the numerical analysis is extended to precisely mimic the experiment, with an inhomogeneous Gaussian pump and a weak wedge potential. It is shown that the inhomogeneous pumping is the cause of the observed asymmetry of excitations.

A. Homogeneous numerical model

We perform a numerical analysis using the truncated Wigner method [5] to simulate the polariton field ψ, with the same microscopic parameters as the experiments. Specifically, we calculate

$$i\hbar d\psi(\mathbf{r},t) = dt\Bigg[-\frac{\hbar^2\nabla^2}{2m} + i\frac{\hbar}{2}\left(\frac{\gamma(\mathbf{r})}{1+\frac{|\psi(\mathbf{r},t)|^2_-}{n_s}} - \kappa\right) + g|\psi(\mathbf{r},t)|^2_- + V(\mathbf{r})\Bigg]\psi(\mathbf{r},t) + \sqrt{\frac{\gamma(\mathbf{r})+\kappa}{4}}dW \quad (1)$$

where dW is the Wiener noise with zero mean satisfying $\langle dW^*(\mathbf{r},t)dW(\mathbf{r}',t)\rangle = 2\delta_{\mathbf{r},\mathbf{r}'}dt$, $m \approx 3.8\cdot 10^{-5}m_e$ is the polariton effective mass, $\gamma(\mathbf{r})$ is the Gaussian pump, $\kappa \approx 1/200\,\text{ps}^{-1}$ is the effective decay rate, and $g = 0.004\,\text{meV}\,\mu\text{m}^2$ and n_s=$1000\,\mu\text{m}^{-2}$ are the effective polariton-polariton interaction strength and the saturation density, respectively. The Wigner commutator contribution has been subtracted from the polariton field density, $|\psi(\mathbf{r},t)|^2_- \equiv |\psi(\mathbf{r},t)|^2 - 1/2\text{dV}$ with dV being the size of the numerical grid, and we take $|\psi(\mathbf{r},t)|^2_- \ll n_s$ as we are at low densities. First, we examine the homogenous case where the potential $V(\mathbf{r}) = 0$ and the pump is uniform $\gamma(\mathbf{r}) = \gamma_0$.

There are two potential sources of noise in this analysis: from drive and decay through the dW term in Eq. (1) and from thermal fluctuations through adding white noise as an initial condition and averaging over different realisations. As we are interested in the way the spectrum is populated rather than the exact occupations of all the modes, and given also that the system is essentially thermal, we present here only the initial condition approach for generating the noise (simulations with the dW term show qualitatively the same behaviour but due to greater background noise the images are not as sharp).

To simulate the equation of motion, Eq. 1, we use the XMDS2 software framework. Specifically, we utilise an adaptive step-size algorithm based on fourth- and fifth-order embedded Runge-Kutta methods with periodic boundary conditions, and perform additional tests to eighth- and ninth-order. The periodic boundary conditions are an artefact of using a Fast Fourier Transform (FFT) to efficiently switch between real and momentum space when evaluating the kinetic energy, which ensures very fast computation of each time step. However, they also allow flux leaving the system to come back in the other side, which we mitigate by implementing an additional absorbing boundary condition where the depth and width are adjusted to the geometry of the experiment.

The numerical integration of the equation of motion is implemented on a 2D finite grid of $N_x = N_y = 1024$ points with grid spacing $l = 1.0\,\mu\text{m}$, where the large size of the simulation box ensures that the polariton density drops practically to zero at the boundary. In order to obtain smooth data, we average over ten initial noise values. By Fourier transforming the polariton wavefunction $\psi(\mathbf{r},t)$ and taking the modulus squared, we find the excitation spectrum for progressively increasing pump powers, which is shown in Supplementary Fig. 3. It can be

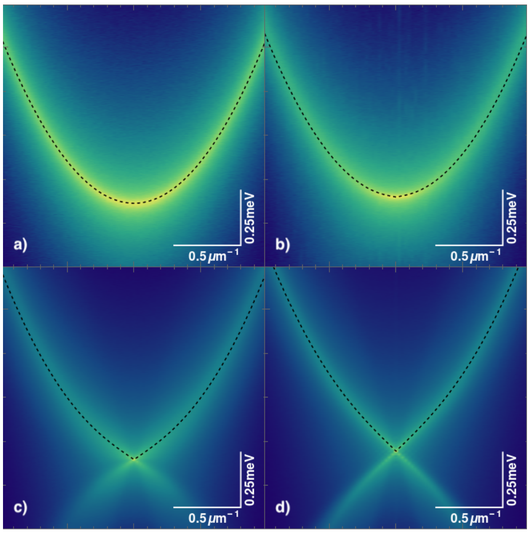

Supplementary Figure 3: Numerical simulations of the excitation spectrum for homogeneous (spatially uniform) pumping. **a**, At low pump powers the spectrum is parabolic (dashed black lines). **b, c,** As the power is gradually increased, there is a crossover to the Bogoliubov spectrum (marked by dashed black lines), which is clearly visible in **d**.

seen that there is a change from a parabolic to a Bogoliubov spectrum as the system transitions to the condensed phase. However, both branches of the Bogoliubov spectrum are clearly visible.

B. Analytical model with finite condensate velocity

To investigate how a finite condensate velocity affects the behaviour of the system, we analytically calculate the PL spectrum of fluctuations in the presence of such a velocity in the Bogoliubov approximation. The retarded Green's function of a weakly interacting bose gas is widely known [6] and we reproduce it here with the addition of an imaginary broadening parameter [7]:

$$D^R(\omega, \boldsymbol{k}) = \frac{1}{d^R(\omega, \boldsymbol{k})} \begin{pmatrix} \hbar\omega + i\hbar\eta + \epsilon(\boldsymbol{k}) + gn_0 & -gn_0 + i\hbar\eta \\ -gn_0 - i\hbar\eta & -\hbar\omega - i\hbar\eta + \epsilon(-\boldsymbol{k}) + gn_0 \end{pmatrix}, \tag{2}$$

$$d^R(\omega, \boldsymbol{k}) = (\hbar\omega + i\hbar\eta - \epsilon(-\boldsymbol{k}) - gn_0)(\hbar\omega + i\hbar\eta + \epsilon(\boldsymbol{k}) + gn_0) + g^2 n_0^2 + (\hbar\eta)^2,$$

where the advanced Green's function is given by the conjugacy relation, $D^A = [D^R]^\dagger$. Here, η is the broadening parameter, n_0 the polariton mean-field density, and the chemical potential is given by $\mu = gn_0$. To include a finite condensate velocity, we introduce the wavevector $\boldsymbol{k}_c$ in the dispersion relation, giving

$$\epsilon(\boldsymbol{k}) = \hbar^2 \frac{(\boldsymbol{k} + \boldsymbol{k}_c)^2 - k_c^2}{2m}, \tag{3}$$

which without loss of generality can be taken to be in the x-direction, $\boldsymbol{k}_c = (k_c, 0)$. The PL is given in thermal equilibrium by [8]

$$L(\omega, \boldsymbol{k}) = \frac{i}{2\pi} n_B(\hbar\omega) \left[D_{11}^R(\omega, \boldsymbol{k}) - D_{11}^A(\omega, \boldsymbol{k}) \right], \tag{4}$$

where $n_B(\hbar\omega) = 1/(\exp(\hbar\omega/k_B T) - 1)$ is the Bose-Einstein distribution. Substituting in the Green's functions, this becomes

$$L(\omega, \boldsymbol{k}) = -\frac{\hbar\eta}{\pi \left| d^R(\omega, \boldsymbol{k}) \right|^2} n_B(\hbar\omega) \left((\hbar\omega)^2 + 2\hbar\omega(\epsilon_+ + gn_0) + \epsilon_+(\epsilon_+ + 2gn_0) + (\hbar\eta)^2 \right) \tag{5}$$

where $\epsilon_\pm = \epsilon(\pm\boldsymbol{k})$. Because we have introduced the broadening, η, to approximate a real system, the divergence in the Bose-Einstein distribution at $\omega = 0$

presents a problem. This can be mitigated by introducing a factor $\nu(\boldsymbol{k}) = -\epsilon(\boldsymbol{k})$, calculated from $D^R_{11}(\nu/\hbar, \boldsymbol{k}) - D^A_{11}(\nu/\hbar, \boldsymbol{k}) = 0$, in the place of the chemical potential. I.e., we calculate

$$L(\omega, \boldsymbol{k}) = \frac{i}{2\pi} n_B(\hbar\omega - \nu(\boldsymbol{k})) \left[D^R_{11}(\omega, \boldsymbol{k}) - D^A_{11}(\omega, \boldsymbol{k})\right], \tag{6}$$

The result of this calculation for the same effective mass and interaction strength as the above simulations, plus $k_c = 0.1\,\mu\text{m}^{-1}$, $n_0 = 10\,\mu\text{m}^{-2}$, $\hbar\eta = 0.003\,25\,\text{meV}$, and $k_B T = 2\,\text{meV}$ is given in Supplementary Fig. 4. Clearly, both branches of the excitation spectrum are visible, suggesting that a finite condensate velocity is not the cause of the experimental observation of only one populated branch.

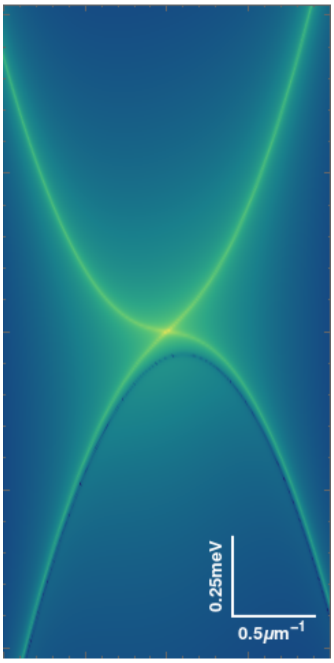

Supplementary Figure 4: The PL spectrum of fluctuations calculated analytically in the Bogoliubov approximation for homogeneous pumping with a uniform current of finite momentum $k_c = 0.1\,\mu\text{m}^{-1}$. Clearly the spectrum is tilted, but unlike the experimental observations both branches of excitations are visible.

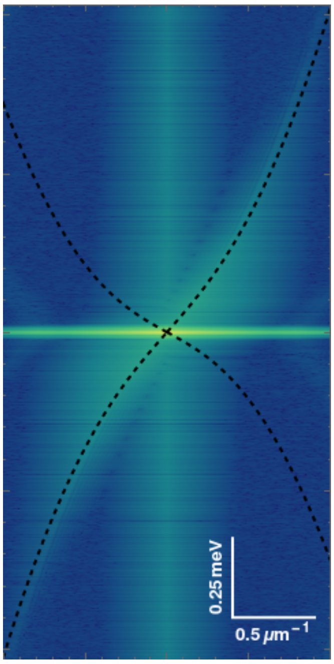

Supplementary Figure 5: The excitation spectrum, under the exact experimental configuration and same microscopic parameters, obtained numerically by Fourier transforming the first order spatiotemporal correlation function $g^{(1)}(\Delta x, \Delta t)$, shown in Eq. (10), with ψ calculated from Eq. (1). The analytical tilted dispersion is shown by the dashed black line.

C. Inhomogeneous numerical model

To simulate the inhomogeneous nature of the experiment, we replace $V(\mathbf{r})$ in Eq. (1) with a weak wedge potential and $\gamma(\mathbf{r})$ with a Gaussian external pump, defined by:

$$V(\mathbf{r}) = \frac{V_0}{L_0}(L - x), \tag{7}$$

$$\gamma(\mathbf{r}) = \gamma_0 e^{\mathbf{r}^2/(2\sigma^2)} \tag{8}$$

where $V_0 = 0.1\,\text{meV}$, $L_0 = 400.0\,\mu\text{m}$ and $\sigma \approx 8.49\,\mu\text{m}$ so FWHM $= 20.0\,\mu\text{m}$. We treat the pump strength, γ_0, as the only tunable parameter.

Using the same computational process as outlined in the previous section for the homogeneous case, we simulate the excitation spectrum using two different methods: first, in a more conventional fashion, by Fourier transforming the polariton wavefunction $\psi(\mathbf{r}, t)$ and taking the modulus squared, and secondly, with the same procedure applied in the experiments, by Fourier transforming the first order spatiotemporal correlation function $g^{(1)}(\Delta x, \Delta t)$, where x is defined along the red line in Fig. 5 of the main text. The spatiotemporal correlation function is defined as

$$g^{(1)}(\Delta x, \Delta t) = \frac{\langle \psi^*(\Delta x, \Delta t)\psi(0,0)\rangle}{\sqrt{\langle|\psi(\Delta x, \Delta t)|^2\rangle\langle|\psi(0,0)|^2\rangle}} \tag{10}$$

where $\langle \ldots \rangle$ indicates an average over the initial noise. A comparison of the results of these two methods is shown in Figs. 5c and 5d in the main text, where the only dif-

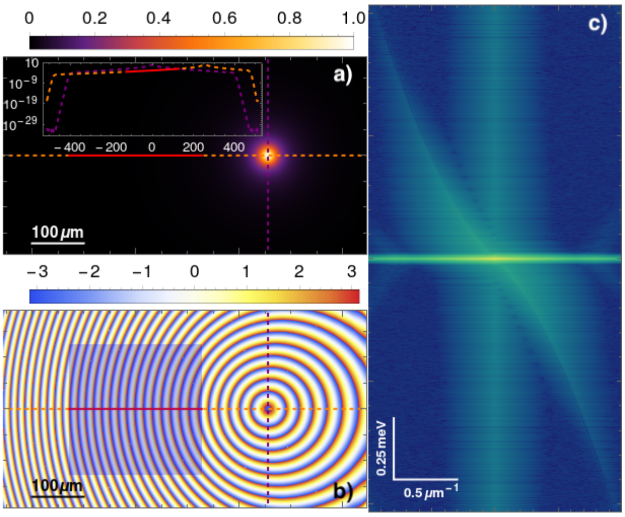

Supplementary Figure 6: Numerical calculations of the excitation spectrum, similar to Fig. 5 in main text but with the pump placed on the right-hand side of the measured region and with an inverted wedge potential. **a,** Density profile of the condensate, both on a linear and (inset) logarithmic scale, where the solid red line corresponds to the measured region. **b,** Phase profile (color scale in radians) of the condensate in 2D space, where the region used to calculate the spectrum is shown by the semitransparent rectangle. **c,** In comparison to Fig. 5 of main text the asymmetric Goldstone dispersion has reversed.

ference is that the ghost branch is less populated for the spectrum obtained from the polariton density.

In Supplementary Fig. 5, the excitation spectrum obtained from the first order spatiotemporal correlation function is shown fitted to the analytical tilted Bogoliubov dispersion given in Eq. 2 of the main text (dashed black line). It can be seen that, with the exception of the missing branch in the numerical excitation spectrum corresponding to backward propagation, the numerical and analytical results perfectly match. Note that the vertical and horizontal occupations are an artificial effect due to periodic boundary conditions. The directionality of the excitations is analogous to the dispersion of surface gravity waves in the presence of wind, in which only one branch can be observed. This effect derives in our case from the asymmetric pumping configuration, where only part of the condensate on the right-hand side of the pumping laser is considered. Within the pump spot where the exciton reservoir is localised, quantum and thermal fluctuations are mostly directed radially outwards. These fluctuations populate the collective excitations of the condensate according to their direction, and thus contribute negligibly to states directed against the "wind". To test this analogy, we have repeated the same numerical calculations where now the measured region is on the left-hand side of the laser spot with an inverted wedge potential. In Supplementary Fig. 6, we show the

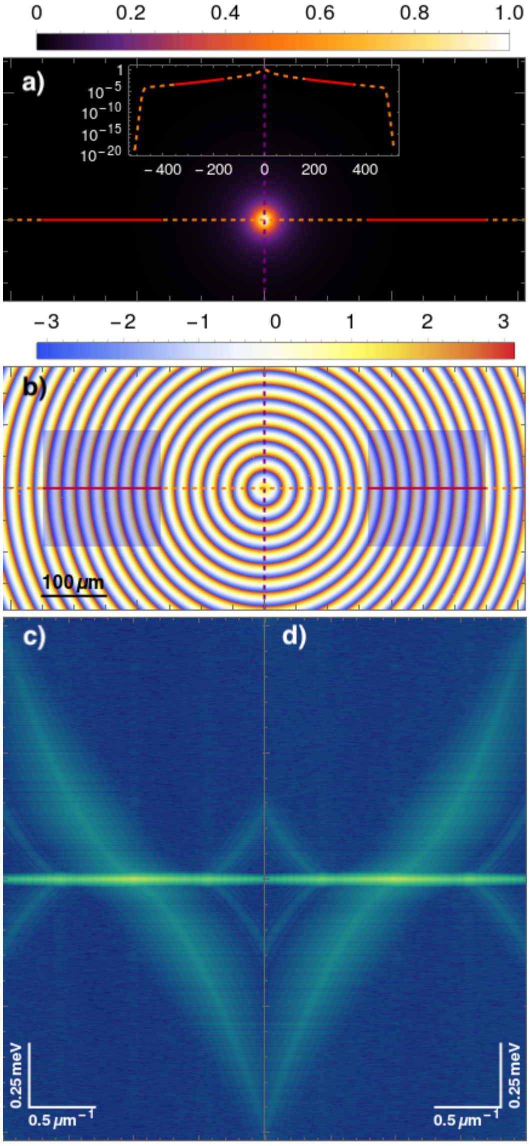

Supplementary Figure 7: Numerical calculation of the excitation spectrum in the absence of the wedge potential. **a,** Density profile of the condensate shown on a linear scale where the regions used to determine the spectra, which are now on both sides of the pump spot, correspond to the solid red lines. Inset: density profile on a logarithmic scale. **b,** Phase profile (colour scale in radians) of the condensate, where the absence of the wedge potential can be seen from the lack of distortion. The regions used to calculate the spectra are indicated by the semitransparent rectangles. **c, d,** Spectra from the left-hand and right-hand regions, respectively. The directionality of the excitation spectrum survives even without the wedge potential. By calculating spectra from both sides of the pump, we see clearly how their position relative to the pump spot determines the observed directionality.

resulting excitation spectrum, which is flipped over with respect to that in Supplementary Fig. 5 as the condensate propagation is reversed.

Finally, in order to investigate the influence of the weak wedge potential on the excitation spectrum, we simulate the case in which this potential is absent, leading to a potential landscape rotationally symmetric with respect to the centre of the laser spot. One can see from Supplementary Fig. 7 that the directionality of the excitation spectrum survives, suggesting that the weak wedge potential is not the determining factor of this effect. Figs. 7c and 7d show the excitation spectra for the left-hand and right-hand measured regions, respectively, supporting the hypothesis that the directionality of the spectrum is caused by fluctuations propagating radially out from the pump spot.

[1] J. P. Dugan, G. J. Fetzer, J. Bowden, G. J. Farruggia, J. Z. Williams, C. C. Piotrowski, K. Vierra, D. Campion, and D. N. Sitter, Journal of Atmospheric and Oceanic Technology **18**, 1267 (2001), ©American Meteorological Society. Used with permission.

[2] H. Lamb, *Hydrodynamics*, Vol. 6th edition Cambridge University Press, (1879) (Dover Publications, New York, 1932).

[3] P. A. Hwang, J. Geophys. Res. **110**, C10029 (2005).

[4] E. Wertz, L. Ferrier, D. D. Solnyshkov, R. Johne, D. Sanvitto, A. Lemaître, I. Sagnes, R. Grousson, A. V. Kavokin, P. Senellart, G. Malpuech, and J. Bloch, Nature Physics **6**, 860 (2010).

[5] P. Comaron, G. Dagvadorj, A. Zamora, I. Carusotto, N. Proukakis, and M. Szymańska, Physical review letters **121**, 095302 (2018).

[6] A. Griffin, *Excitations in a Bose-condensed liquid*, Vol. 4 (Cambridge University Press, Cambridge, 1993).

[7] G. Roumpos, M. Lohse, W. H. Nitsche, J. Keeling, M. H. Szymańska, P. B. Littlewood, A. Löffler, S. Höfling, L. Worschech, A. Forchel, *et al.*, Proceedings of the National Academy of Sciences **109**, 6467 (2012).

[8] F. Marchetti, J. Keeling, M. Szymańska, and P. Littlewood, Physical Review B **76**, 115326 (2007).
